# Special Wonders of the Canal

**DOI:** 10.3201/eid2708.AC2708

**Published:** 2021-08

**Authors:** Byron Breedlove

**Affiliations:** Centers for Disease Control and Prevention, Atlanta, Georgia, USA

**Keywords:** art science connection, emerging infectious diseases, art and medicine, about the cover, the conquerors (Culebra Cut, Panama Canal), Jonas Lie, special wonders of the canal, Panama Canal, Culebra Cut, public health, malaria, parasites, protozoa, yellow fever, viruses, mosquitoes, vector-borne infections, Aristides Agramonte, James Carroll, David du Bose Gaillard, William C. Gorgas, Carlos Finlay, Jesse Lazear, Walter Reed, Ronald Ross

**Figure:**
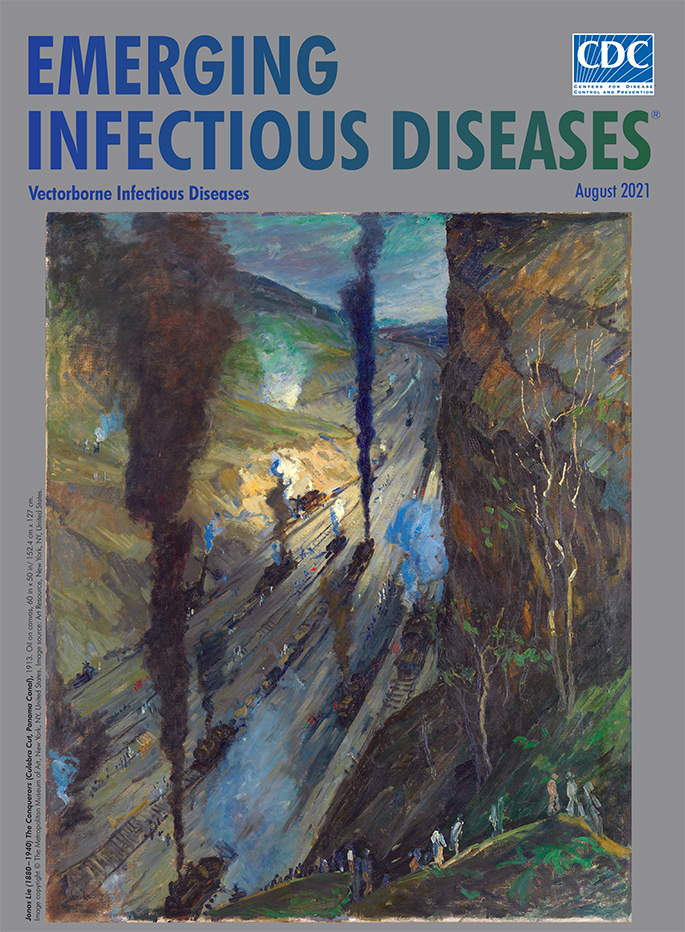
**Jonas Lie (1880−1940). *The Conquerors (Culebra Cut, Panama Canal)*, 1913.** Oil on canvas, 60 in x 50 in/152.4 cm x 127 cm. Image copyright © The Metropolitan Museum of Art, New York, NY, USA. Image source: Art Resource, New York, NY, USA.

The official opening of the Panama Canal in August 1914 marked the culmination of an idea that Charles V, Holy Roman Emperor and King of Spain, proposed in 1534, two decades after Spanish explorer Vasco Nunez de Balboa had visited the Isthmus of Panama. After an attempt by France to forge a canal connecting the Atlantic and Pacific Oceans was abandoned―in large measure because yellow fever and malaria devastated its workforce―the United States arranged to continue the work in 1904. The US undertaking was enormous and lasted another decade, moved enough earth and rubble to create a 16-foot-wide tunnel reaching the center of the Earth, and was deemed by the American Society of Civil Engineers to be among the 7 greatest civil engineering achievements of the 20th century. 

The Culebra Cut,^1^ a 9-mile stretch through the Continental Divide from Gamboa on the Chagres River in the north to Pedro Miguel in the south between Bas Obispo and Pedro Miguel, proved to be the most challenging section to finish, and this project was dubbed “the special wonder of the canal.” Historian David McCullough notes the Culebra Cut “was the great focus of attention, regardless of whatever else was happening at Panama. The building of Gatun Dam or the construction of the locks, projects of colossal scale and expense, were always of secondary interest so long as the battle raged in that nine-mile stretch.” 

Construction in the cut continued day and night under unforgiving conditions and involving as many as 6,000 workers, including Panamanians, West Indians, and African Americans. Blistering tropical heat that could reach up to 120°F was compounded by a rainy season that lasted 9 months and by miserably high humidity. Reverberating off the canyon walls was an unrelenting cacophony created by up to 300 drills, 60 to 70 shovels each loading several trains, whistles and shouts from the workers, and countless explosions. McCullough writes, “For seven years Culebra Cut was never silent, not even for an hour.” Deadly landslides occurred without warning, becoming larger and more frequent as work progressed and destroying machinery, burying workers, and reversing months of momentum. Deaths caused by trains, construction equipment, falls, and explosions occurred daily. 

The Isthmus of Panama was also a perfect environment for mosquitoes. However, because the mosquitoborne illnesses of yellow fever and malaria that waylaid the effort by France had been controlled by the time work started on the cut, the Panama Canal was eventually completed ahead of schedule and under budget. 

Credit centers on the work of Colonel William C. Gorgas, who after his successful efforts to control yellow fever in Cuba in 1901 was appointed chief sanitation officer for the Panama Canal project in 1904. He and his team of sanitary engineers enacted strictly enforced, integrated measures―including draining sources of standing water, applying larvicides, and screening windows―that virtually eliminated yellow fever and greatly reduced the toll of malaria, diseases that Carlos Finlay and Ronald Ross, respectively, had only a few years earlier shown were transmitted by mosquitoes. Gorgas drew upon the pioneering work of Walter Reed, James Carroll, Aristides Agramonte, and Jesse Lazear (Lazear died of yellow fever in Cuba in September 1900), who had elucidated the role of the mosquito in yellow fever transmission. They paved the way for Gorgas to implement the assiduous and extraordinarily effective prevention/control program. According to Centers for Disease Control and Prevention, the malaria death rate for employees dropped from 11.59 per 1,000 in November 1906 to 1.23 per 1,000 in December 1909, and deaths from malaria in the total population decreased from 16.21 per 1,000 in July 1906 to 2.58 per 1,000 in December 1909.

­ Near the end of 1912 artist Jonas Lie viewed―and was captivated by―an early color movie, *The Making of the Panama Canal*. In early 1913, Lie made a 3-month sojourn to Panama, where he completed an estimated 30 paintings of the work. Lie witnessed the final stages of work as the excavation of Culebra Cut was completed on May 20, 1913. According to the Hudson River Museum, Lie “was enthralled by the feats of engineering required to dig the Culebra Cut, as well as the sublime visual qualities of the massive trench being carved across the Isthmus of Panama. Working tirelessly in the intense tropical heat, he produced oil sketches and drawings and took careful notes on the technical aspects of the canal construction.” Lie’s *The Conquerors (Culebra Cut, Panama Canal),* featured on this month’s cover, is among the best-known depictions of the canal’s construction and the most celebrated painting from his excursion. 

Although the painting is interspersed with earth tones, flecks of red, and smudges of green, the tones that dominate the palette are blue, black, gray, and white. Along the gorge’s floor, coal-fired locomotives traverse parallel train tracks, belching black plumes of smoke that rise like sooty geysers and churning hazy clouds of bluish steam into the humid air. The bulging slope of the cut on the left, its facets captured in bold strokes, and a sheer vertical rock wall on the right, provide scale and perspective. Vestiges of native flora clinging to the right wall offer a reminder that this area was once verdant. Workers trudge the steep path from the bottom, engulfed by their surroundings. Bartholomew F. Bland, deputy director of the Hudson River Museum, says of *The Conquerors*: “It looks like hell, like an inferno . . . there’s all this black smoke.” 

During the effort to build the canal in the 1880s, more than 22,000 workers from France died, many from malaria and yellow fever, before the etiologies of those tropical diseases were understood. Records indicate that during the period of US construction, more than 55,000 people were employed and an estimated 5,600 died of injury and disease. The death toll would have been higher without effective protocols to control vectorborne diseases, in effect a second “special wonder of the canal.” Many of those practices Gorgas instituted continue to be important in global efforts to control mosquitoborne illnesses.

## Footnotes

1 From 1915 to 2000, this section of the canal was named the Gaillard Cut after US Major David du Bose Gaillard, who had supervised much of the construction. After the United States transferred control of the canal to Panama In 2000, the name was changed back to the Culebra Cut (its original name from 1914−1915). Culebra is also the name of the mountain ridge that this artificial valley cuts through.
